# Metabolic Regulation of Mycobacterial Growth and Antibiotic Sensitivity

**DOI:** 10.1371/journal.pbio.1001065

**Published:** 2011-05-24

**Authors:** Seung-Hun Baek, Alice H. Li, Christopher M. Sassetti

**Affiliations:** 1 Department of Microbiology and Physiological Systems, University of Massachusetts Medical School, Worcester, Massachusetts, United States of America; 2 Howard Hughes Medical Institute, Chevy Chase, Maryland, United States of America; Harvard University, United States of America

## Abstract

Treatment of chronic bacterial infections, such as tuberculosis (TB), requires a remarkably long course of therapy, despite the availability of drugs that are rapidly bacteriocidal in vitro. This observation has long been attributed to the presence of bacterial populations in the host that are “drug-tolerant” because of their slow replication and low rate of metabolism. However, both the physiologic state of these hypothetical drug-tolerant populations and the bacterial pathways that regulate growth and metabolism in vivo remain obscure. Here we demonstrate that diverse growth-limiting stresses trigger a common signal transduction pathway in *Mycobacterium tuberculosis* that leads to the induction of triglyceride synthesis. This pathway plays a causal role in reducing growth and antibiotic efficacy by redirecting cellular carbon fluxes away from the tricarboxylic acid cycle. Mutants in which this metabolic switch is disrupted are unable to arrest their growth in response to stress and remain sensitive to antibiotics during infection. Thus, this regulatory pathway contributes to antibiotic tolerance in vivo, and its modulation may represent a novel strategy for accelerating TB treatment.

## Author Summary

Despite the availability of antibiotics that rapidly kill bacteria in vitro, the treatment of chronic bacterial infections, such as tuberculosis, requires long-term drug therapy. The reasons for this are unclear, but many have hypothesized that the slow replication and concomitantly low metabolic rate of bacteria in the host environment produce an “antibiotic-tolerant” state. We have tested this hypothesis by identifying the bacterial pathways responsible for slowing the growth and metabolism of *Mycobacterium tuberculosis* in response to stress. We found that diverse growth-limiting stresses trigger a common signal transduction pathway that slows bacterial growth by redirecting cellular carbon fluxes away from central metabolic pathways and towards storage. Disruption of this metabolic switch increased the antibiotic sensitivity of the bacterium during infection, verifying that this response significantly contributes to antibiotic tolerance and suggesting new strategies for accelerating therapy.

## Introduction

Over fifty years after the discovery of antimycobacterial drugs, *Mycobacterium tuberculosis* remains an endemic pathogen throughout much of the world. Based on immunological tests, one-third of the global population has been exposed to this organism, which sickens 10 million and kills 2 million yearly [1]. Arguably, the most important factor limiting TB control efforts is the remarkably long antibiotic regimen that is necessary to eradicate the pathogen [2]. Despite the availability of drugs that rapidly kill the bacterium in vitro, treatment with these agents requires at least 6 mo. Incomplete treatment is both ineffective and promotes the selection of drug-resistant strains.

The reasons that antibiotics are less effective in vivo remain unclear but likely reflect the altered metabolic state of the bacterium in this environment [3]. In the mammalian host, *M*. *tuberculosis* is challenged by a variety of pressures, including low oxygen, iron limitation, low pH, and changes in nutrient availability [4]–[7]. In vitro, many bacteria respond to similar environmental stresses by arresting their growth and assuming a quiescent or dormant state in which they remain viable until the environment once again becomes favorable [8]. Similarly, *M. tuberculosis* dramatically reduces both its growth and metabolic activity in chronically infected animals, doubling only once every 100 h or more [9],[10]. Since virtually all antibiotics preferentially kill rapidly replicating bacteria [3],[11], it has been hypothesized that the reduced growth and metabolic activity of these quiescent populations is responsible for the “antibiotic-tolerance” observed during infection [12].

While the physiologic state of these slowly replicating mycobacterial populations in vivo is difficult to investigate directly, in vitro models have been developed to mimic this condition. The best defined of these models is long-term hypoxic culture, which has been proposed to mimic the oxygen tension found in some TB lesions [13]. When *M. tuberculosis* is cultured under oxygen-limiting conditions, this obligate aerobe ceases replicating and adopts an antibiotic-tolerant state that can be maintained almost indefinitely [14],[15]. While macromolecular synthesis slows dramatically during this period, continual ATP production is required for survival, indicating that cellular metabolism remains at least nominally active [13].

Taken together, these observations indicate that *M. tuberculosis* is able to adopt a relatively quiescent antibiotic-tolerant state both in vitro and within the host. Previous efforts to eradicate nonreplicating bacterial populations have generally focused on the development of drugs that directly kill these organisms. As an alternative to this approach, we sought to define the bacterial functions that govern mycobacterial growth and could therefore be manipulated to increase drug sensitivity. In this work, we define a functional pathway that enables the bacterium to reduce its metabolic rate in response to environmental stress. Mutants lacking this regulatory pathway remain markedly more sensitive to antibiotics during infection, demonstrating that this specific response contributes to the antibiotic tolerance observed in vivo.

## Results

### Identification of Growth-Regulatory Pathways

To understand the mechanisms controlling the growth of *M. tuberculosis* during infection, we sought to identify mutants that had lost the ability to arrest their growth and continued to replicate in hypoxic culture. We subjected a library of transposon mutants to a low oxygen environment sufficient to arrest the growth of wild type *M. tuberculosis* [6] and used transposon site hybridization [16] to identify the set of mutants that were overrepresented after 6 wk of culture, suggesting a growth or survival advantage.

**Figure 1:**
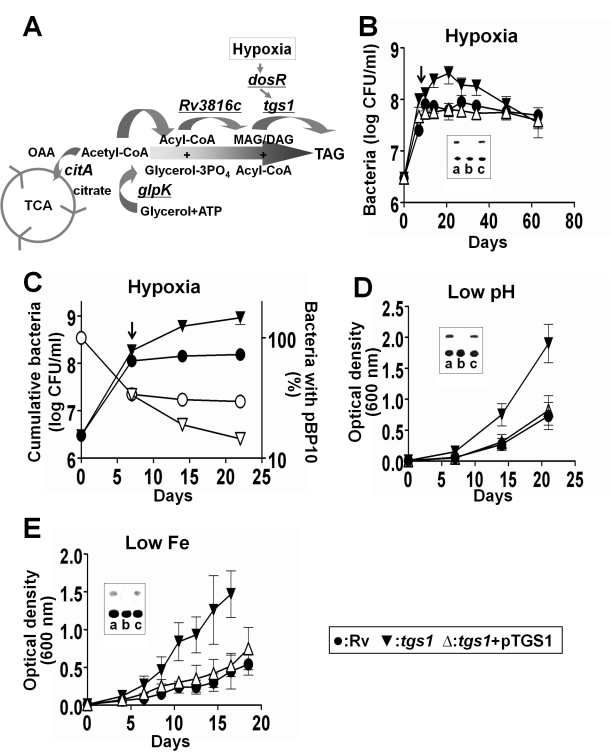
Triglyceride synthesis mutants continue to replicate under growth-limiting conditions. (A) The predicted TAG biosynthetic pathway of *M. tuberculosis* and its relationship to the TCA cycle. Mutations in the underlined genes were predicted by Transposon Site Hybridization to result in overrepresentation after hypoxia. OAA, oxaloacetate; MAG, monoacylglycerol; DAG, diacylglycerol. (B) Δ*tgs1* bacteria grow to a higher cell density in hypoxic cultures. (C) Δ*tgs1* mutants continue to replicate in hypoxic culture. The replication dynamics of the indicated strains were assessed by quantifying the rate at which unstable plasmid pBP10 was lost (right axis, open symbols). The “cumulative bacterial number” (left axis, closed symbols) represents the total number of organisms that would have been present if cell death was negated. Arrows in (B) and (C) indicate the initiation of hypoxia based on methylene blue decolorization. (D and E) Growth of *M. tuberculosis* strains at an initial pH of 5.5 (D) and in low iron medium (E). Optical density measurements are shown (similar data were obtained by quantifying CFU). Means ± SD of two independent experiments each performed in duplicate or triplicate are shown. Insets demonstrate the lack of TAG accumulation (upper species) in Δ*tgs1* bacteria, as assessed by thin layer chromatography. Each TLC was developed independently. In inset, “a,” H37Rv; “b,” Δ*tgs1*; and “c,” complemented strain Δ*tgs1*+*pTGS1*.

Prominent among the 34 identified genes (Table S1) were several predicted to encode the enzymes necessary to produce triacylglycerol (TAG) from glycerol and acyl-CoA (Figure 1A). The gene that appeared to play the most important role, *tgs1*, encodes a well-characterized TAG synthase that represents the dominant triglyceride synthetic activity under hypoxia [17],[18]. The importance of the *tgs1* gene under this condition is likely due to its transcriptional induction via the DosR regulator, which controls the earliest response to hypoxia [19]. Consistent with the known regulatory relationship between *tgs1* and DosR, our genetic screen also indicated that *dosR* mutants were overrepresented in the library exposed to hypoxia. The similar phenotypes of mutants lacking virtually every step in this pathway indicated that DosR-triggered TAG accumulation was critical for hypoxia-induced growth arrest.

To verify the predictions of our genetic screen, we generated a mutant lacking the *tgs1* gene. In aerated broth cultures, both wild type and Δ*tgs1* mutant bacteria accumulated only small amounts of TAG and grew at similar rates (unpublished data). However, when these bacteria were cultured in sealed vials to produce hypoxia [15], the Δ*tgs1* mutant failed to accumulate the TAG observed in wild type or complemented mutant cells and grew to a density that was 10-fold higher than wild type (Figure 1B). To confirm that the Δ*tgs1* mutant continued to replicate under hypoxia, we calculated the doubling time of the bacteria by quantifying the segregation of an unstable plasmid that was lost at a constant rate per cell division [9]. Indeed, while wild type bacteria completely arrested growth once hypoxia was established, the Δ*tgs1* mutant continued to replicate for at least 14 more days (Figure 1C). Even between 14 and 21 d, when the total number of Δ*tgs1* bacteria did not change significantly, the cells continued to segregate the plasmid. This confirmed that the Δ*tgs1* strain was unable to arrest its growth, and even the apparent stasis of this strain represented a state of balanced growth and death. While the cause of death remains uncertain, the cytosolic ATP concentration of the mutant decreased as oxygen was consumed (Figure S2), indicating that replication in the absence of this preferred electron acceptor produced an untenable metabolic state.

*M. tuberculosis* accumulates TAG under a variety stresses, including hypoxia, iron limitation, and low pH [17],[20],[21], indicating that TAG synthesis might modulate growth under multiple conditions. Indeed, we found that while each of these conditions retarded the growth of wild type bacteria and the complemented mutant, the Δ*tgs1* strain continued to grow at a relatively rapid rate (Figure 1D–E). While the DosR regulon was known to be induced during hypoxia [19], this regulator had not been shown to act in these other conditions. To determine if the same regulatory circuit was operational, we used a reporter derived from the promoter of the well-characterized DosR target, *acr* [22]. We found that this promoter was strongly induced under low iron conditions and weakly induced by low pH (Figure S3). Induction under these conditions is likely due to the recently described activation of DosR by alterations in cellular redox state [23]. Despite this difference in degree of induction, we found that a mutant lacking the *dosR* gene behaved similarly to the Δ*tgs1* in each condition, indicating that this sensor kinase was important for all of these responses.

### TAG Synthesis Inhibits Growth by Reducing TCA Flux

When *M. tuberculosis* or related environmental bacteria are exposed to stress, they accumulate large cytosolic stores of triglycerides [20],[24]. This dramatic production of lipid suggested that the growth regulatory effects of *tgs1* induction might be due to the wholesale redirection of carbon flux into TAG synthesis and away from intermediary metabolic pathways. Since acetyl CoA is a primary substrate of both the TCA cycle and TAG synthesis, we hypothesized that TAG production lowered the growth and metabolic rate of the organism by directly competing for this metabolite (see Figure S1).

**Figure 2:**
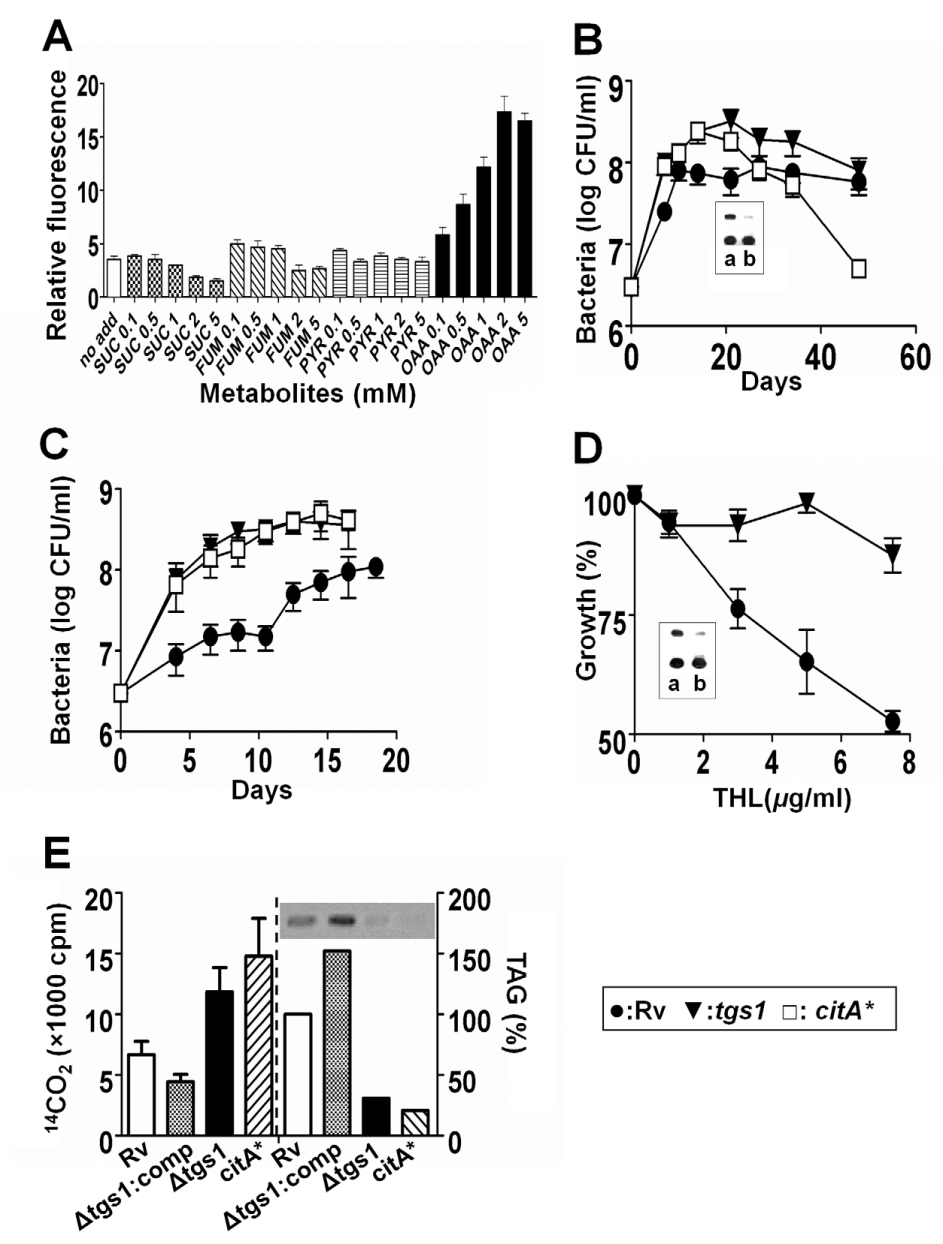
TAG synthesis modulates growth by consuming acetyl CoA. (A) Oxaloacetate (OAA) stimulates bacterial growth in low iron medium. Growth of H37Rv expressing *gfp* was assessed in 384-well plates by fluorometry. Wells contained medium alone, succinate (SUC), fumarate (FUM), pyruvate (PYR), or oxaloacetate (OAA). Each metabolite was added at increasing concentrations (0.1, 0.5, 1, 2, and 5 mM). Fluorescence intensity of the plates was measured after 10 d of growth and normalized to control wells containing a Sybr green standard. (B and C) Growth of the indicated strains was assessed in hypoxic (B) or low iron cultures (C). “citA*” indicates citrate synthase overexpressing strain. The inset in (B) shows TAG accumulation by H37Rv (a) and the citA* strain (b) under hypoxic conditions. (D) Addition of tetrahydrolipostatin (THL) to low iron cultures inhibits growth in a *tgs1*-dependent manner. Growth inhibition was determined from the optical density cultures after 21 d. Means ± SD of two independent experiments each performed in duplicate or triplicate are shown. The inset shows TAG accumulation by H37Rv (a) and the *tgs1* strain (b) with the highest tested concentration of THL. Lipid extracts were normalized to represent the same bacterial mass. (E) Radiolabeled acetate (^14^C_1,2_) was introduced into hypoxic vials after 7 d of sealed culture. The left four bars indicate the cpm of CO_2_ sampled from the headspace after 6 h. The right four bars indicate relative abundance of ^14^C-labeled TAG on the same time point. Inset shows a representative TLC plate that was quantified. Means ± SD of triplicate experiments are shown for CO_2_ measurements.

Acetyl CoA is incorporated into the TCA cycle by citrate synthase, which condenses it with oxaloacetate (OAA) to form citrate (Figure 1A). To test whether TAG synthesis competes with citrate synthase for acetyl CoA, we supplied wild type *M. tuberculosis* with exogenous OAA, which we expected to promote citrate synthase activity by increasing substrate concentration. As anticipated, this treatment mimicked the Δ*tgs1* mutation by enhancing growth in both hypoxic and iron-restricted cultures (Figures 2A, S4). Other related metabolites had no effect on growth, supporting the conclusion that OAA enhances growth under these conditions by stimulating citrate synthase activity.

Similarly, we tested this model by overexpressing the *citA* gene, encoding citrate synthase. This excess enzyme activity appeared to effectively compete for acetyl CoA, as the overexpression strain (citA*) resembled the Δ*tgs1* mutant in both hypoxic and low iron culture; that is, bacteria continued to grow and failed to accumulate TAG (Figure 2B,C). Both *citA* overexpression and oxaloacetate addition in hypoxic conditions appeared to have an even more pronounced effect than *tgs1* deletion, as the viability of these cultures decreased more rapidly than the Δ*tgs1* strain once oxygen was depleted.

To verify that titrating the flux between these two competing pathways produced the expected changes in growth rate, we employed a small molecule inhibitor of TAG degradation. The accumulation of TAG is antagonized by cellular lipases that release the acyl chains for degradation (Figure S1). Thus, we expected that inhibiting this reverse, lipase-dependent, pathway would promote carbon accumulation in TAG and thereby inhibit growth under stress. To test this prediction, we added tetrahydrolipostatin (THL), a broad-spectrum lipase inhibitor, to bacteria cultured under conditions that induce TAG synthesis and retard growth. As predicted, the addition of THL caused a dose-dependent decrease in the growth of wild type, but not *tgs1*-deficient, bacteria (Figures 2D and S5).

Finally, to directly demonstrate that the TCA cycle and TAG synthesis compete for the same carbon pool, we quantified the relative rates of carbon flux into these two pathways by metabolic labeling with [^14^C]-acetate. Under hypoxic conditions, we found that the deletion of *tgs1* and overexpression of *citA* had a similar effect. Both manipulations increased acetate flux into CO_2_ via the TCA cycle, at the expense of TAG production (Figure 2E). The antagonistic effect of TAG synthesis on TCA flux was independently verified by monitoring the abundance of amino acids, which represent relatively stable markers of the TCA activity. We found that the intracellular concentrations of lysine, threonine, and alanine, amino acids derived from oxaloacetate or affected by its turnover, were decreased in wild type bacteria as they lowered their metabolic activity during adaptation to hypoxia (Figure S6). In contrast, deletion of *tgs1* or overexpression of *citA* reversed this decline, verifying that TCA activity remained relatively high in these strains. In sum, the opposing effects of the Tgs1 and CitA enzymes on both growth and carbon flux indicate that TAG production restricts the growth of wild type bacteria by diverting carbon away from growth-promoting pathways such as the TCA cycle.

### Modulation of Carbon Fluxes Can Reverse Antibiotic Tolerance In Vitro and In Vivo

**Figure 3:**
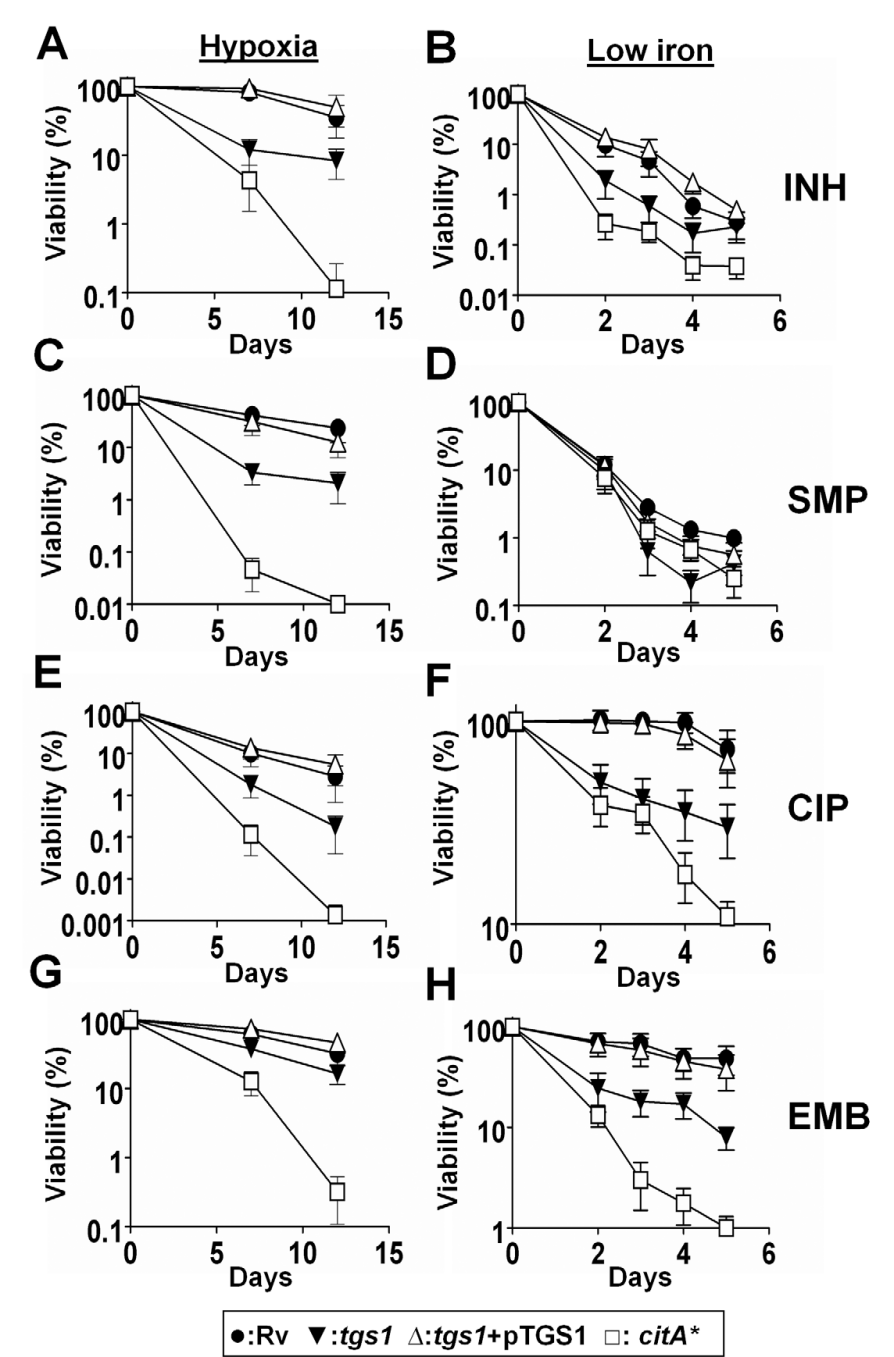
Metabolic modulation reverses the antibiotic tolerance induced by low iron and hypoxic conditions. Bacterial survival in the presence of the indicated antibiotics under hypoxic conditions (A, C, E, G) and in low iron media (B, D, F, H). Isoniazid (“INH”, 2 and 0.25 µg ml^−1^, A and B), streptomycin (“SMP”, 2 and 1 µg ml^−1^, C and D), ciprofloxacin (“CIP”, 4 and 1 µg ml^−1^, E and F), and ethambutol (“EMB”, 5 and 3 µg ml^−1^, G and H) were introduced into each culture. Antibiotics were added to the hypoxic vials after 14 d of culture. Means ± SD of two independent experiments each performed in duplicate are shown.

Since decreased metabolic activity generally correlates with lower antibiotic efficacy, we speculated that TAG synthesis might contribute to the drug-tolerant phenotype induced by stress. We tested this hypothesis using in vitro conditions that trigger TAG accumulation. Indeed, we found that the Δ*tgs1* mutant remained significantly more sensitive to a variety of antibiotics under tolerance-inducing conditions such as hypoxia and iron limitation (Figure 3). The antibiotics used were chemically distinct and targeted diverse cellular pathways, suggesting that the general hypersensitivity of the Δ*tgs1* bacteria was due to a fundamental alteration in cellular metabolism. As expected, the increased multidrug-susceptibility of the Δ*tgs1* mutant was much less pronounced under favorable growth conditions in which this gene is not induced (Figure S7). Under these conditions, the mutant was no more susceptible than wild type to any of the drugs tested, except the fatty acid synthesis inhibitor, isoniazid (INH). We conclude that while TAG synthesis may influence INH sensitivity through multiple mechanisms, the multidrug susceptibility of the Δ*tgs1* mutant is due to a general increase in growth rate and/or metabolic activity. This conclusion was supported by the remarkable antibiotic sensitivity of the citA* strain that we observed in tolerance-inducing cultures (Figure 3). This strain was killed even more rapidly than the Δ*tgs1* mutant, verifying that metabolic rate is a major determinant of antibiotic susceptibility under these conditions.

**Figure 4:**
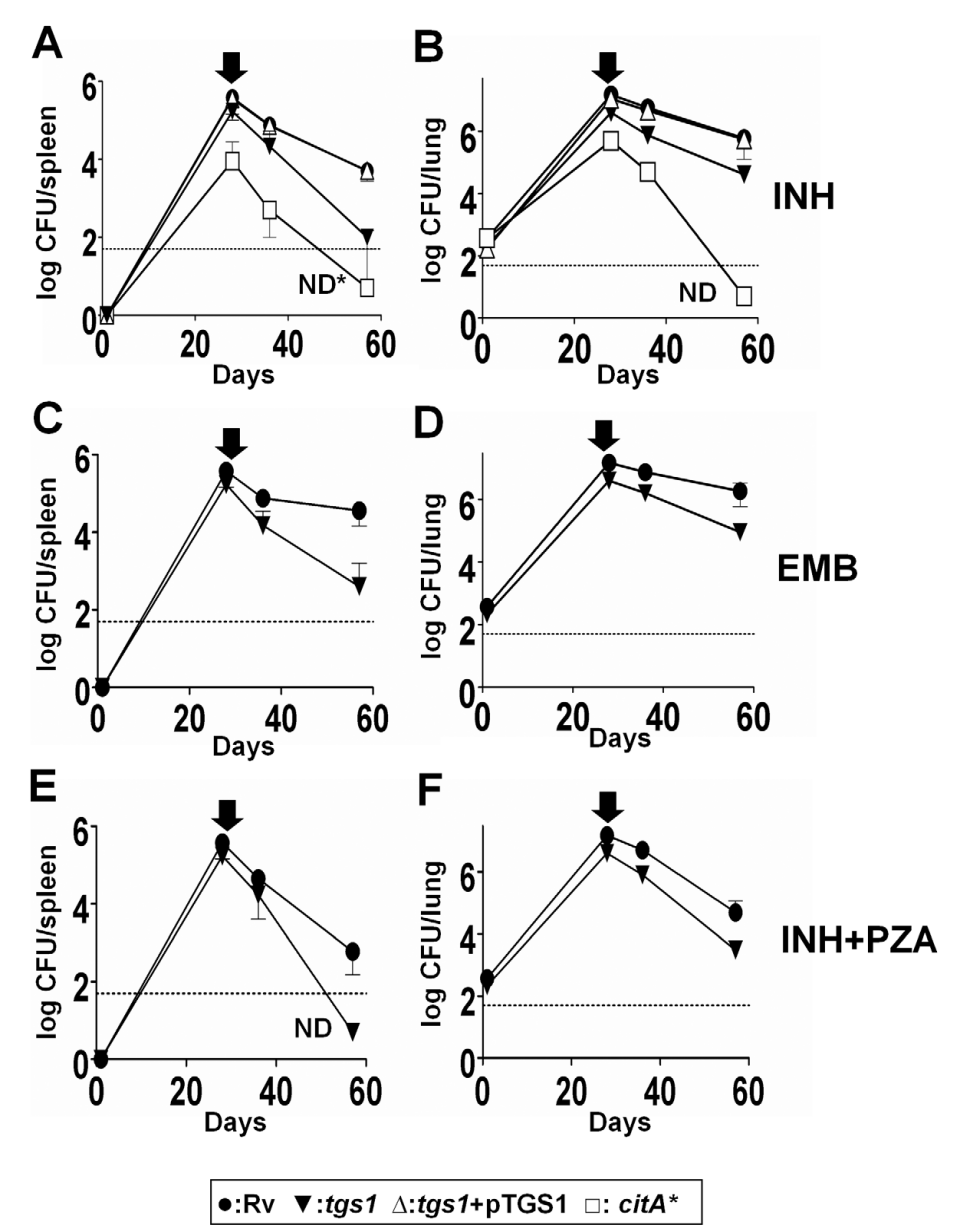
Modulating carbon fluxes reverses the antibiotic tolerance induced during infection. Mice were infected via the aerosol route with the indicated bacterial strains. Total bacterial burden in the spleens (A, C, E) and lungs (B, D, F) is shown. Mice were treated at the indicated times with isoniazid (“INH”, A, B), ethambutol (“EMB”, C, D), or isoniazid plus pyrazinamide (“INH+PZA”, E, F). Dotted line represents the detection limit of the experiment. “ND” indicates no colonies detected. ND* indicates two colonies were detected but neither retained the *citA* overexpression plasmid. Means ± SD from three to five mice are shown.

Induction of the *tgs1* gene and TAG accumulation occur during infection [20], and TCA activity appears to be limited in this environment [25]. Therefore, we next investigated whether TCA limitation by TAG synthesis was also required for antibiotic tolerance in vivo. The Δ*tgs1* mutation did not overtly disrupt the physiology of the bacterium in vivo, as only subtle defects in bacterial viability were observed in mice infected with the mutant (Figure S8). Despite this apparently normal behavior, the metabolic state of the mutant was clearly different from wild type, as the Δ*tgs1* strain remained significantly more sensitive to several antibiotic regimens targeting different cellular functions (Figure 4). Consistent with a central role for TCA activity in antibiotic tolerance in vivo, we found that overexpressing citrate synthase had a more pronounced effect. The citA* strain displayed a modest growth or survival defect in mice (Figures 4A,B and S8), indicating that increased TCA flux under these conditions decreased overall fitness. More importantly, this strain remained even more sensitive to antibiotics during infection than the Δ*tgs1* mutant, as we had previously observed under in vitro stress conditions. After 28 d of monotherapy, the number of viable wild bacteria had only decreased 20-fold, while the number of viable *citA* overexpressors was reduced below the limit of detection (Figure 4A,B).

## Discussion

Many organisms accumulate TAG in preparation for long periods of inactivity. Previously, this response had been largely thought to serve a carbon storage function, allowing the rapid restoration of metabolism upon resuscitation [18]. More recently, it has been proposed that TAG synthesis may be important for redox homeostasis in cells with low respiratory activity [26]. In addition to these potential functions, we now show that TAG synthesis represents an active stress response that can play a causal role in governing growth, metabolic rate, and antibiotic susceptibility by redirecting cellular carbon fluxes (Figure S1).

Reducing metabolic rate in response to stress is likely to be advantageous for a variety of reasons. In the most general terms, continual growth under conditions lacking a critical nutrient or cofactor can result in catastrophic imbalances in cellular metabolism, as we observed in the hypoxia model. While the Δ*tgs1* and citA* strains have a temporary fitness advantage over wild type bacteria in hypoxia, these mutants are unable to sustain this advantage due to an increased rate of cell death. A similar failure to reduce metabolic activity under growth-limiting stress also resulted in the attenuation of the citA* strain in vivo.

An additional important consequence of the low metabolic rate of *M. tuberculosis* during infection is decreased antibiotic sensitivity. We found that the redirection of carbon into TAG synthesis was critical for assuming this antibiotic tolerant phenotype under a variety of different in vitro and in vivo stresses. As anticipated, antibiotic sensitivity was correlated with replication rate under many conditions, but this correlation was not absolute. For example, increased replication could not account for the heightened susceptibility of the citA* strain relative to the Δ*tgs1* mutant in vitro, as both strains appeared to grow similarly at the time of treatment. Replication rate alone was also unlikely to account for the hypersensitivity of these strains in vivo. We did not observe increased numbers of viable bacteria in the tissues of mice infected with Δ*tgs1* or citA* strains and were unable to detect progressive histopathology or the accumulation of bacterial chromosomes by quantitative PCR (unpublished data) that would be anticipated if continual growth and death were occurring [10]. Thus, we conclude that the increased metabolic activity (i.e., TCA flux) of these strains reversed the general antibiotic tolerance induced in vivo, even though replication was effectively suppressed by the combination of stresses produced by host immunity. Many antibiotics kill bacteria not by inhibiting a specific cellular target but by producing toxic metabolic byproducts [27]–[29]. Most of these products, particularly reactive oxygen species, are produced in a TCA-dependent manner in antibiotic-treated bacteria [27], suggesting that the drug sensitivity of the more metabolically active Δ*tgs1* and citA* strains was likely due to the increased production of toxic intermediates.

Despite the central role played by the *tgs1* gene in restricting growth under a number of environmental stresses, the induction of this gene is certainly not the only mechanism regulating metabolic rate in response to stress. The observation that citrate synthase overexpression has a quantitatively larger effect than *tgs1* deletion suggests that this enzyme may compete with multiple redundant acetyl CoA-consuming pathways of which Tgs1-mediated TAG synthesis is only one. Indeed, we identified a second TAG synthase in our genetic screen (Table S1) that is also likely to contribute to metabolic regulation. In addition, mycobacteria are known to accumulate glycogen upon nitrogen starvation [30], and increased flux into the gluconeogenic pathway could also consume acetyl CoA and limit metabolism. Thus, while *tgs1* plays an indispensable role in limiting mycobacterial growth under the DosR-stimulating conditions described here, it is likely that other pathways contribute under different conditions. A number of additional genes, such as those encoding both succinate- and pyruvate dehydrogenase, were identified in our screen (Table S1). Further study will be required to determine if these enzymes also act by redirecting carbon fluxes or if distinct mechanisms are responsible.

The propensity for mycobacteria to accumulate TAG in response to stress has been described previously [20],[31], but the physiological role of this response has remained unclear. Our work demonstrates that TAG synthesis represents an active stress response in *M. tuberculosis* that promotes antibiotic tolerance both in vitro and in vivo by reducing growth and metabolic activity. This supports the hypothesis that quiescent bacterial populations are responsible for the relative inefficacy of antibiotics in vivo. We suggest that the manipulation of these metabolic regulatory pathways might represent a novel strategy to improve antibiotic efficacy, once the consequences of such an intervention on pathogenesis and the acquisition of drug resistance are more thoroughly understood.

## Materials and Methods

### Bacterial Strains, Plasmids, and Culture Conditions

*Mycobacterium tuberculosis* H37Rv (ATCC 27294) and *Escherichia coli* DH5α were used. For aerated culture, *M*. *tuberculosis* (Mtb) was grown in Middlebrook 7H9 broth (Difco) supplemented with 0.05% Tween-80 and ADC enrichment, or on 7H10 agar with 10% OADC enrichment (Becton Dickinson) at 37°C. Hygromycin and kanamycin were added at 50 and 25 µg/ml, respectively. All cultures including aerated and TAG-accumulating cultures (below) were initiated at 2.5×10^6^ CFU/ml. For low pH culture, 7H9 broth was adjusted to pH 5.5 with 0.1 N HCl. When necessary, tetrahydrolipstatin (THL), from 20 mg/ml stock in methanol, was added. For low iron culture, Sauton's media with 0.05% Tween-80 was mixed with 20 g/l Chelex (BioRad). The chelated solution was sterile filtered and supplemented with MgSO_4_ (4.2 mM) and FeCl_3_ (0.1 µM). The inocula was washed with 10 µM EDTA for 10 min and then washed twice with iron-free PBS containing 0.05% Tween-80.

For hypoxic cultures, bacteria were inoculated into 17 ml of 7H9 broth supplemented with Tween-80 and ADC in a 25 ml screw cap vial, which was sealed with a teflon/silicon screw cap (Wheaton) and parafilm. Cultures were agitated using a small magnetic stir bar rotating at 100–150 rpm/min. At a specific time point, two or three vials were opened and viable bacterial numbers were enumerated on 7H10 agar.

For measurement of replication during hypoxia, H37Rv and Δ*tgs1* strains carrying plasmid pBP10 [32] were inoculated into hypoxic culture, as described above. The percentage of mycobacteria carrying the plasmid and theoretical doubling time were determined as described [9].

For bacterial culture in 384-well plates, inocula of H37Rv carrying pMSP12::GFP [33] were prepared in 7H9 broth as described above and dispensed into a 384-well plate (25 µl of culture per well) containing low iron media. The relevant metabolic intermediates were added to each well at final concentrations of 0.1, 0.5, 1, 2, 5, and 10 mM. Plates were incubated at 37°C. Fluorescence was quantified using a plate reader. Designated wells containing PBS + Tween-80 and 10 nM of SYBR Green dye (Bio-Rad) were used to normalize between readings.

### Genetic Manipulation of Mtb

The *tgs1* gene (nucleotide #'s 3497344-3494008, as annotated at http://genolist.pasteur.fr/TubercuList/) was replaced by a hygromycin-resistance marker using the pJM1 suicide plasmid, as described [34]. For complementation, the open reading frame (ORF) of *tgs1* including 167 bps upstream nucleotides encompassing the putative promoter was cloned into the integrating plasmid pMV306, and the resulting plasmid was transformed into *M*. *tuberculosis*. The *dosR* deletion mutant was generously provided by Dr. David Sherman. To constitutively express *citA* (Rv0889c), the *citA* ORF was cloned into pAL5000-based plasmids, pUV15tetO and pMV261. The strain bearing pUV15tetO::citA was used for all presented data. However, all results were confirmed using the strain harboring pMV261::citA. The empty vector pUV15tetO had no effect on Mtb growth.

### Transposon Site Hybridization

Two independent libraries of 10^5^ *himar*-1 transposon mutants were seeded (OD_600_ of 0.02) into 50 ml conical tubes containing 35 ml of 7H9 medium including Tween-80 and OADC. Cultures were agitated as described above at 37°C for 6 wk, and oxygen consumption was verified by the decolorization of methylene blue. After selection, the surviving mutants were recovered by plating on 7H10 agar in parallel with the initial library. Hypoxic and control pools were then compared in duplicate using TraSH, essentially as described [35]. Mutants that were significantly overrepresented after hypoxic culture were defined using the following criteria: arbitrary fluorescence intensity >300 in one of the two channels, fluorescence ratio (hypoxic/control) >3, and *t* test *p* value <0.05 after false testing correction (GeneSpring GX. Agilent).

### Drug Treatment In Vitro and Biochemical Analysis

Isoniazid (INH, Sigma), Ethambutol (EMB, Sigma), Streptomycin-sulfate (SMP, Sigma), and Ciprofloxacin (CIP, Bayer) were used. Indicated concentrations of drug were added at the initiation of aerated and low iron cultures or injected at 14 d of post-incubation into hypoxic cultures using a gas-tight syringe (Hamilton). At each time point, the bacterial viability in two to three independent cultures was quantified by washing bacteria twice in PBS + Tween-80 and plating.

To analyze TAG content, bacteria were washed in PBS two times, and total cellular lipids were extracted with chloroform:methanol (2:1). The lipid extracts were dried and redissolved in the same solvent. TAG from bacterial cells, corresponding to 2×10^7^ CFU, was resolved by thin-layer chromatography using glass-baked 250-µm-thick silica gel plates (Whatman) using toluene and acetone (99:1) or hexane and diethylether (9:1) as a solvent. TAG was stained by cerium molybdate and visualized after heating.

Bacterial ATP concentrations of hypoxic cultures were measured using the BacTiter-Glow kit (Promega). Adonosine 5-triphospate disodium (Sigma) was used as a standard.

Relative amino acid levels were measured by Ultrahigh Performance Liquid Chromatography/Electrospray Ionization Tandem Mass Spectrometry with quadruplicate samples of bacterial extracts from 10^9^ cells, as described in [36].

For metabolic flux determination, 2 µCi of 1,2-^14^C-acetate (American Radiolabeled Chemicals) was injected into hypoxic vials at 7 d and the vials were incubated for 1–6 h. The amount of ^14^CO_2_ in the headspace of each vial was measured using a BACTEC TB-460 (Becton Dickinson Co.). CPM increased linearly for the first 6 h after acetate addition. All data shown were sampled during this period. TAG was quantified using a phosphorimager (Fuji Film BAS-2500) following TLC separation.

### Infections and Drug Therapy

C57BL/6 mice were infected through the aerosol route with Mtb at 200–300 CFUs/lung using a Glas-col aerosol exposure system. At the indicated time points, groups of three to five untreated mice were sacrificed, the lungs and spleens were homogenized in PBS containing 0.05% Tween-80, and dilutions were plated on 7H10 agar to enumerate CFU. The indicated groups of mice were treated with antibiotics beginning at 4 wk of postinfection. Drug was delivered *ad libitum* by adding the following concentrations to drinking water: 100 µg/ml INH, 600 µg/ml EMB, and 600 µg/ml Pyrazinamide (PZA, MP Biomedicals). All drug-containing water was replaced weekly. Water consumption was monitored to determine the delivered daily dose (INH: 26.5±0.9 mg/kg, PZA and EMB: 132.6±4.7 mg/kg). No significant difference in consumption was observed between groups. To measure CFU in drug-treated mice, the bacteria in organ homogenates were pelleted by centrifugation and washed with PBS containing 0.05% Tween-80 before plating.

## Supporting Information

Figure S1 Competing acetyl CoA utilizing pathways modulate growth and antibiotic sensitivity in *M. tuberculosis*. Under favorable growth conditions, nutritional carbon is efficiently incorporated into central metabolic pathways, such as the TCA cycle, fueling growth by providing the cell with energy and biosynthetic precursors. Under these conditions, the bacterium is sensitive to antibiotics, which preferentially target rapidly metabolizing cells. A variety of environmental stresses trigger expression of the DosR regulon, leading to the expression of the *tgs1* gene and the conversion of mono- and di-acylglycerol (“MAG” and “DAG”) into TAG. This response redirects the flow of carbon away from growth-promoting pathways and into fatty acid synthesis, effectively retarding the growth and metabolic activity of the organism. Under these conditions, the low growth and metabolic activity of the organism renders it relatively insensitive, or “tolerant” to antibiotics. Genetically manipulating the flux of carbon between these two competing pathways alters both the growth rate and antibiotic sensitivity of *M. tuberculosis*.
(TIF)

Figure S2 Δ*tgs1* mutants are unable to maintain energy homeostasis during inappropriate growth under hypoxia. Graph shows the amount of ATP extracted from bacteria that were cultured for the indicated times in sealed vessels. Means ± SD of two independent experiments each performed in duplicate are shown.
(TIF)

Figure S3 Δ*dosR* and Δ*tgs1* mutants show similar growth phenotypes under stress. Mutants lacking either of these genes were cultured in low iron (A) or low pH (B). Means ± SD of replicate cultures are shown. (C) Relative *acr* promoter activity was determined using an *acr*-luciferase reporter (p*acr-lux* [22]). Log phase aerobically grown bacteria (“O_2_”) are compared with bacteria cultured in low pH media, low Fe media, or in hypoxic culture. Asterisks indicate a significant difference from the “O_2_” sample (* *p* < 0.05, ** *p* < 0.01).
(TIF)

Figure S4 Oxaloacetate transiently enhances viability under hypoxic conditions. Oxaloacetate (“OAA”) was introduced at 7 d into hypoxic cultures. Viable cell numbers increased initially and thereafter declined. Means ± SD of two independent experiments each performed in duplicate are shown (* *p* < 0.05).
(TIF)

Figure S5 Addition of tetrahydrolipostatin (THL) to low iron and pH cultures inhibits growth of H37Rv in a *tgs1*-dependent manner. As indicated in Materials and Methods, a variety of concentrations of THL was added to low iron and pH media at the initiation of culture. Each data point represents the average of triplicate cultures.
(TIF)

Figure S6 Intracellular amino acid abundance indicates that Δ*tgs1* and citA* strains remain metabolically active in hypoxia. The relative abundance of the indicated amino acids in whole cell extracts was determined by liquid chromatography followed by mass spectrometry. Wild type H37Rv in log phase aerobic growth or after 2 wk of hypoxic culture (open or dotted bars, respectively) are compared with hypoxic cultures of the Δ*tgs1* or citA* strains (black or hashed bars, respectively). Measurements are the average of quadruplicate cultures. Values are expressed relative to the hypoxic sample, and asterisks indicate a significant difference from this sample (* *p* < 0.05, ** *p* <0.01).
(TIF)

Figure S7 Δ*tgs1* mutant is not hypersensitive to most drugs under favorable growth conditions. The indicated strains were treated with isoniazid (“INH”, 0.25 µg ml^−1^, A), streptomycin (“SMP”, 1 µg ml^−1^, B), ciprofloxacin (“CIP”, 1 µg ml^−1^, C), or ethambutol (“EMB”, 1 µg ml^−1^, D) for the indicated times and bacterial survival was monitored by plating. Means ± SD of two independent experiments each performed in duplicate are shown.
(TIF)

Figure S8 Effect of modulating carbon fluxes on the growth and survival of *M. tuberculosis* in untreated mice. C57BL/6 mice were infected via the aerosol route with the indicated bacterial strains. Total bacterial burden in the lungs (A) and spleen (B) are shown. Means ± SD from three to five mice are shown. These data are representative of two independent experiments.
(TIF)

Table S1 Mutants found to be overrepresented after hypoxic culture. Replicate libraries of transposon mutants were subjected to 6 wk of culture in sealed vials and compared to the initial pool using transposon site hybridization. Mutants that were significantly overrepresented (criteria are described in the Materials and Methods section) are presented.
(XLS)

## Abbreviations

ATP: Adenosine triphosphate
CoA: coenzyme A
CFU: colony forming unit
CPM: counts per minute
EMB: ethambutol
INH: isoniazid
OAA: oxaloacetate
ORF: open reading frame
PBS: phosphate buffered saline
PCR: polymerase chain reaction
PZA: pyrazinamide
TAG: triacylglycerol (“triglyceride”)
TB: tuberculosis
TCA: tricarboxylic acid cycle
THL: tetrahydrolipostatin
