# Supplementary figures and images for: Metabolic Regulation of Mycobacterial Growth and Antibiotic Sensitivity
Source: PLoS Biol. 2011 May 24;9(5):e1001065. doi: 10.1371/journal.pbio.1001065 (PMC3101192; doi:10.1371/journal.pbio.1001065)

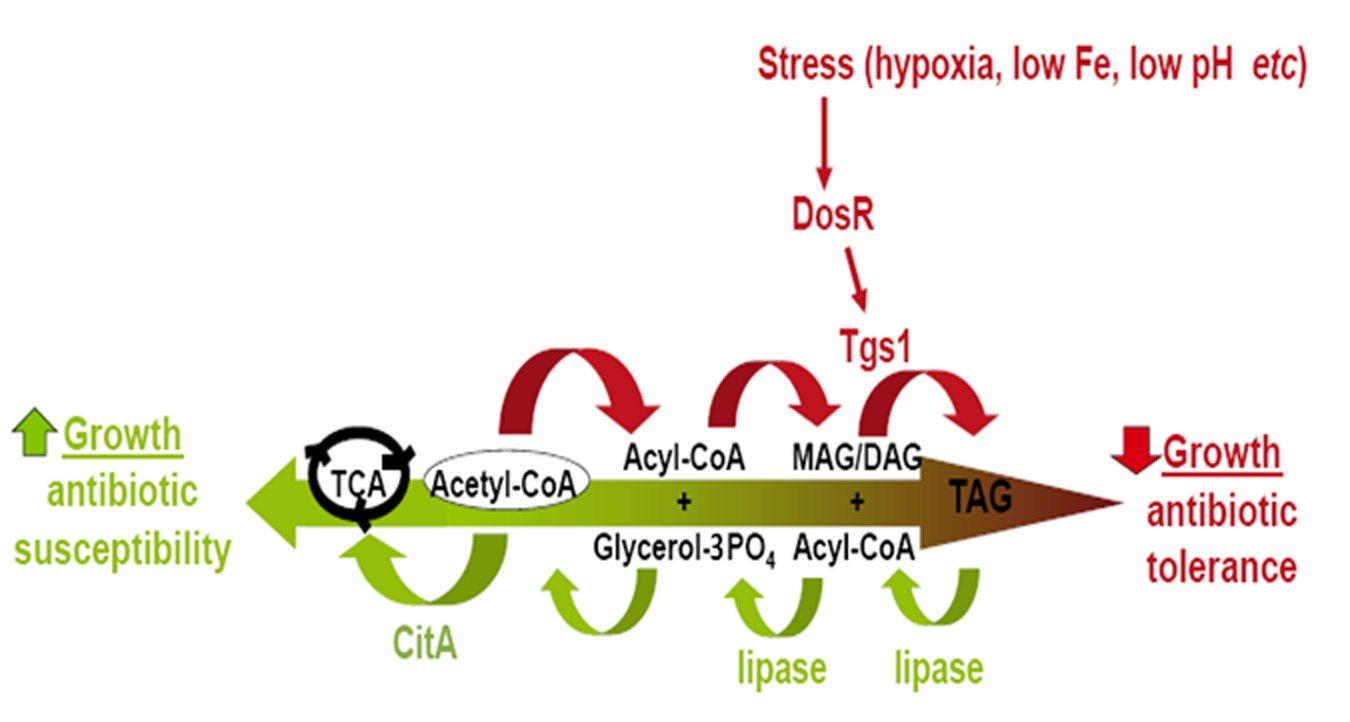

Supplement: Figure S1 — Competing acetyl CoA utilizing pathways modulate growth and antibiotic sensitivity in M. tuberculosis. Under favorable growth conditions, nutritional carbon is efficiently incorporated into central metabolic pathways, such as the TCA cycle, fueling growth by providing the cell with energy and biosynthetic precursors. Under these conditions, the bacterium is sensitive to antibiotics, which preferentially target rapidly metabolizing cells. A variety of environmental stresses trigger expression of the DosR regulon, leading to the expression of the tgs1 gene and the conversion of mono- and di-acylglycerol (“MAG” and “DAG”) into TAG. This response redirects the flow of carbon away from growth-promoting pathways and into fatty acid synthesis, effectively retarding the growth and metabolic activity of the organism. Under these conditions, the low growth and metabolic activity of the organism renders it relatively insensitive, or “tolerant” to antibiotics. Genetically manipulating the flux of carbon between these two competing pathways alters both the growth rate and antibiotic sensitivity of M. tuberculosis. (TIF) [file pbio.1001065.s001.tif]

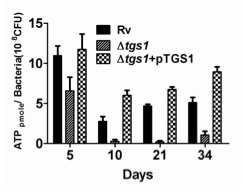

Supplement: Figure S2 — Δtgs1 mutants are unable to maintain energy homeostasis during inappropriate growth under hypoxia. Graph shows the amount of ATP extracted from bacteria that were cultured for the indicated times in sealed vessels. Means ± SD of two independent experiments each performed in duplicate are shown. (TIF) [file pbio.1001065.s002.tif]

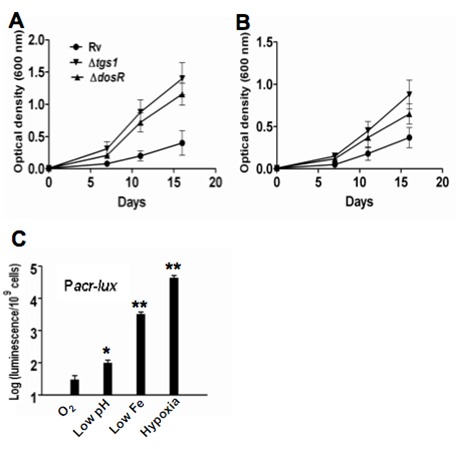

Supplement: Figure S3 — ΔdosR and Δtgs1 mutants show similar growth phenotypes under stress. Mutants lacking either of these genes were cultured in low iron (A) or low pH (B). Means ± SD of replicate cultures are shown. (C) Relative acr promoter activity was determined using an acr-luciferase reporter (pacr-lux [22]). Log phase aerobically grown bacteria (“O2”) are compared with bacteria cultured in low pH media, low Fe media, or in hypoxic culture. Asterisks indicate a significant difference from the “O2” sample (* p < 0.05, ** p < 0.01). (TIF) [file pbio.1001065.s003.tif]

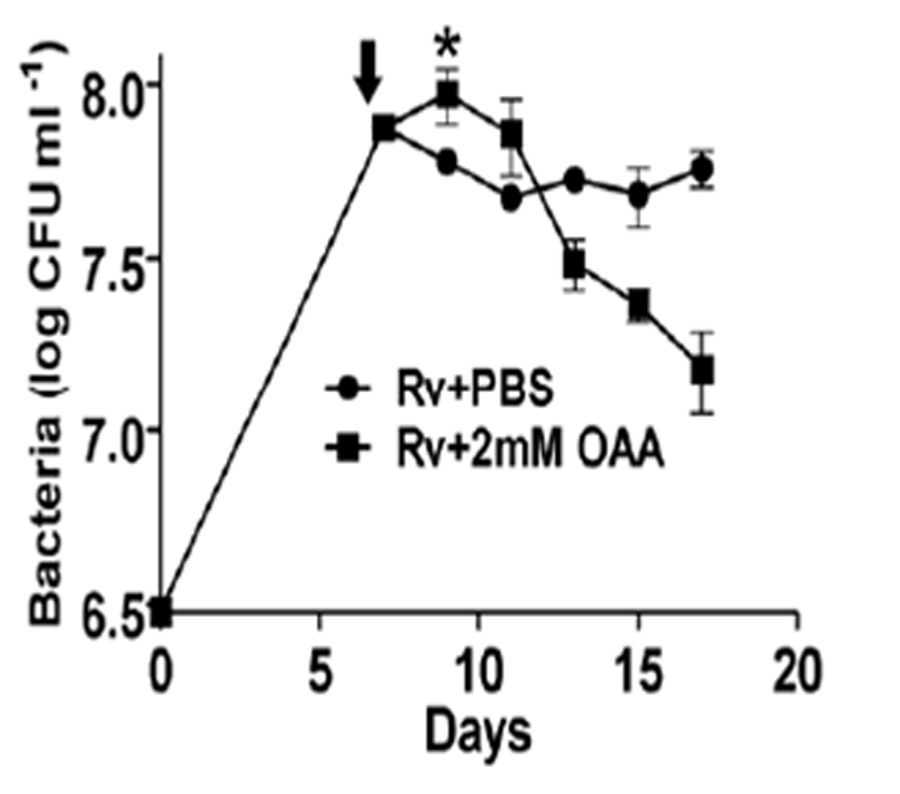

Supplement: Figure S4 — Oxaloacetate transiently enhances viability under hypoxic conditions. Oxaloacetate (“OAA”) was introduced at 7 d into hypoxic cultures. Viable cell numbers increased initially and thereafter declined. Means ± SD of two independent experiments each performed in duplicate are shown (* p < 0.05). (TIF) [file pbio.1001065.s004.tif]

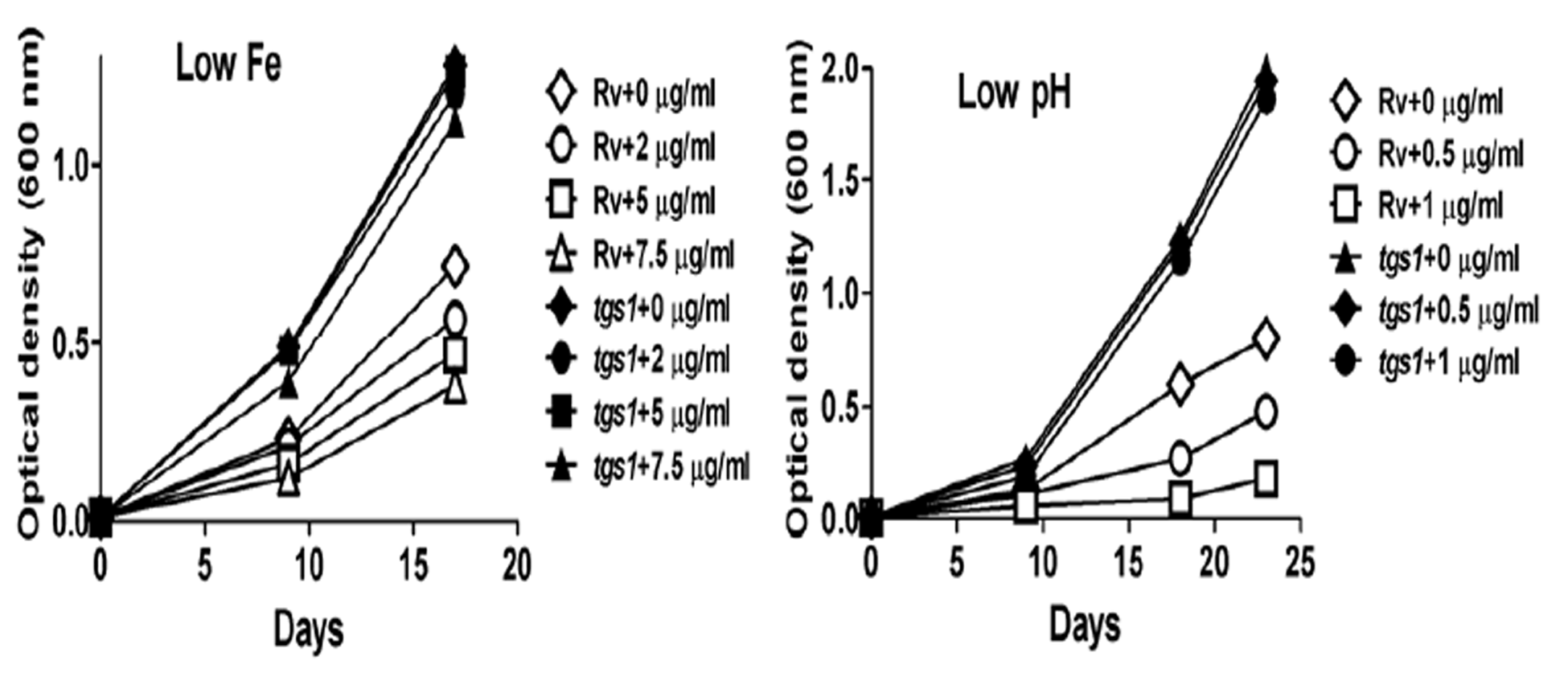

Supplement: Figure S5 — Addition of tetrahydrolipostatin (THL) to low iron and pH cultures inhibits growth of H37Rv in a tgs1-dependent manner. As indicated in Materials and Methods, a variety of concentrations of THL was added to low iron and pH media at the initiation of culture. Each data point represents the average of triplicate cultures. (TIF) [file pbio.1001065.s005.tif]

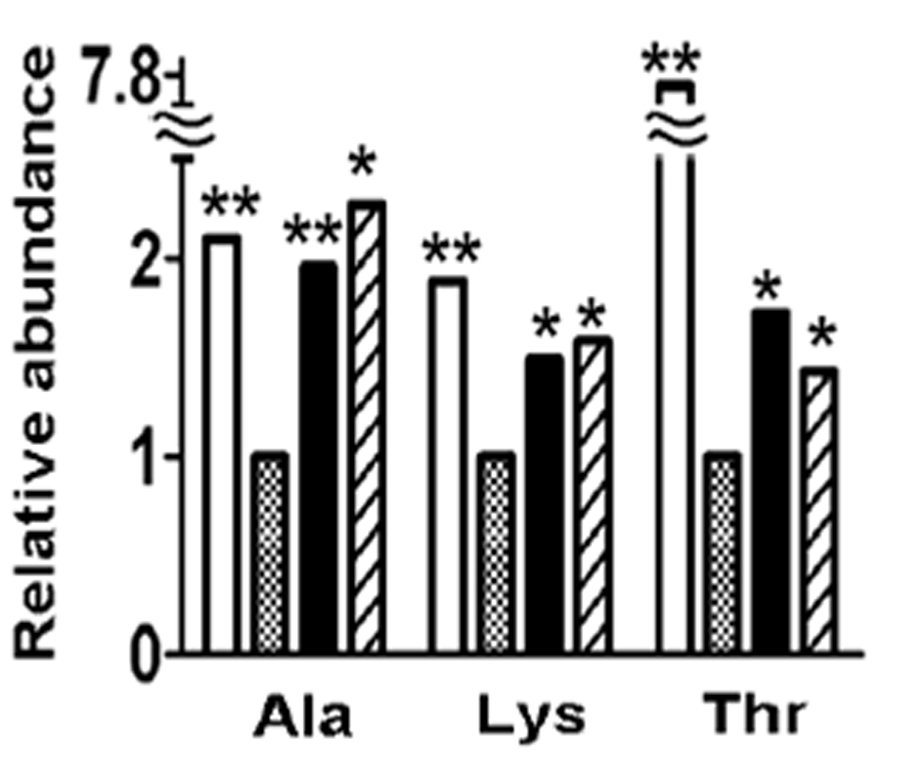

Supplement: Figure S6 — Intracellular amino acid abundance indicates that Δtgs1 and citA* strains remain metabolically active in hypoxia. The relative abundance of the indicated amino acids in whole cell extracts was determined by liquid chromatography followed by mass spectrometry. Wild type H37Rv in log phase aerobic growth or after 2 wk of hypoxic culture (open or dotted bars, respectively) are compared with hypoxic cultures of the Δtgs1 or citA* strains (black or hashed bars, respectively). Measurements are the average of quadruplicate cultures. Values are expressed relative to the hypoxic sample, and asterisks indicate a significant difference from this sample (* p < 0.05, ** p <0.01). (TIF) [file pbio.1001065.s006.tif]

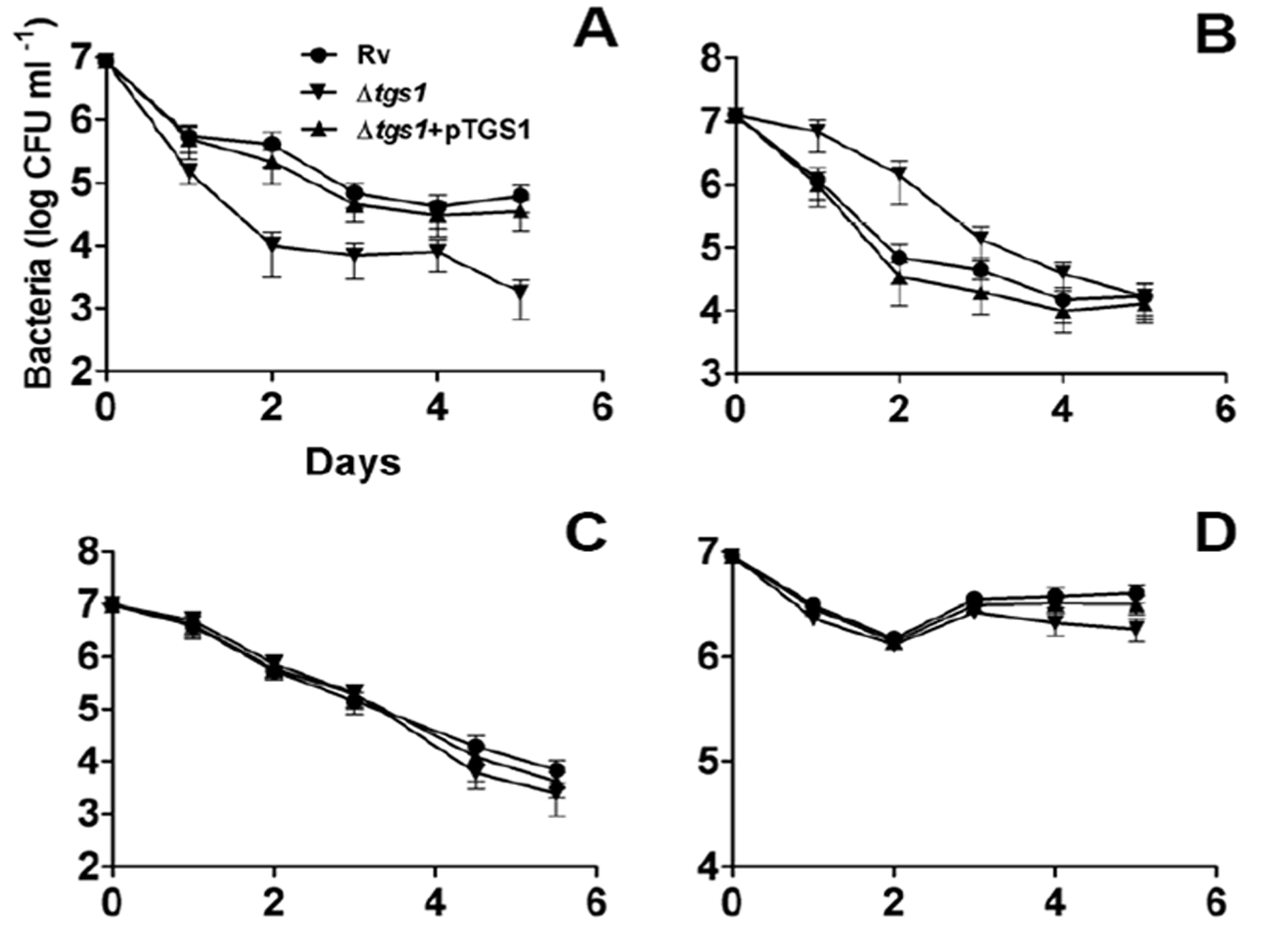

Supplement: Figure S7 — Δtgs1 mutant is not hypersensitive to most drugs under favorable growth conditions. The indicated strains were treated with isoniazid (“INH”, 0.25 µg ml−1, A), streptomycin (“SMP”, 1 µg ml−1, B), ciprofloxacin (“CIP”, 1 µg ml−1, C), or ethambutol (“EMB”, 1 µg ml−1, D) for the indicated times and bacterial survival was monitored by plating. Means ± SD of two independent experiments each performed in duplicate are shown. (TIF) [file pbio.1001065.s007.tif]

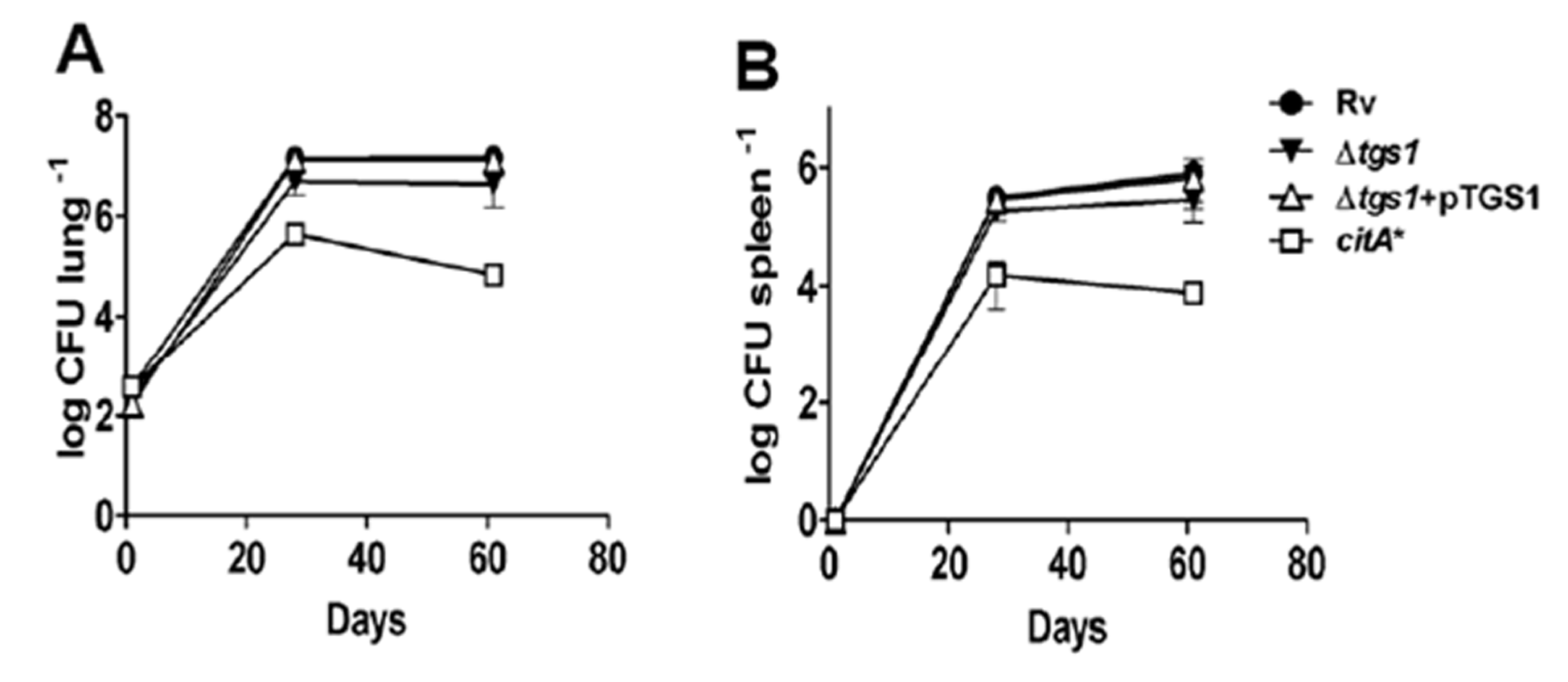

Supplement: Figure S8 — Effect of modulating carbon fluxes on the growth and survival of M. tuberculosis in untreated mice. C57BL/6 mice were infected via the aerosol route with the indicated bacterial strains. Total bacterial burden in the lungs (A) and spleen (B) are shown. Means ± SD from three to five mice are shown. These data are representative of two independent experiments. (TIF) [file pbio.1001065.s008.tif]
